# Genetic Characterization of Brain Metastases in the Era of Targeted Therapy

**DOI:** 10.3389/fonc.2017.00230

**Published:** 2017-09-25

**Authors:** Catherine H. Han, Priscilla K. Brastianos

**Affiliations:** ^1^Departments of Neurology and Radiation Oncology, Division of Hematology/Oncology, Massachusetts General Hospital Cancer Center, Harvard Medical School, Boston, MA, United States; ^2^Auckland Cancer Society Research Centre, Faculty of Medical and Health Sciences, School of Medical Sciences, University of Auckland, Auckland, New Zealand

**Keywords:** brain metastases, genomics, targeted therapy, sequencing, cancer heterogeneity

## Abstract

In the current era of molecularly targeted therapies and precision medicine, choice of cancer treatment has been increasingly tailored according to the molecular or genomic characterization of the cancer the individual has. Previously, the clinical observation of inadequate control of brain metastases was widely attributed to a lack of central nervous system (CNS) penetration of the anticancer drugs. However, more recent data have suggested that there are genetic explanations for such observations. Genomic analyses of brain metastases and matching primary tumor and other extracranial metastases have revealed that brain metastases can harbor potentially actionable driver mutations that are unique to them. Identification of genomic alterations specific to brain metastases and targeted therapies against these mutations represent an important research area to potentially improve survival outcomes for patients who develop brain metastases. Novel approaches in genomic testing such as that using cell-free circulating tumor DNA (ctDNA) in the cerebrospinal fluid (CSF) facilitate advancing our understanding of the genomics of brain metastases, which is critical for precision medicine. CSF-derived ctDNA sequencing may be particularly useful in patients who are unfit for surgical resection or have multiple brain metastases, which can harbor mutations that are distinct from their primary tumors. Compared to the traditional chemotherapeutics, novel targeted agents appear to be more effective in controlling the CNS disease with better safety profiles. Several brain metastases-dedicated trials of various targeted therapies are currently underway to address the role of these agents in the treatment of CNS disease. This review focuses on recent advances in genomic profiling of brain metastases and current knowledge of targeted therapies in the management of brain metastases from cancers of the breast, lung, colorectum, kidneys, and ovaries as well as melanoma.

## Introduction

Brain metastases are the most common central nervous system (CNS) tumors in adults but represent an unmet need in current oncologic practice. The reported incidence of brain metastases is 9–17%; however, the true incidence in the current era of modern cancer therapies is thought to be higher (1). The incidence of brain metastases is rising due, in part, to improved diagnostic techniques and increased patient survival through advanced systemic treatment approaches. Some types of cancers have greater tropism of metastasizing to the brain with most common cancers being lung cancer (39–56%), breast cancer (13–30%), melanoma (6–11%), gastrointestinal cancers (3–8%), and renal cell carcinoma (2–4%) (2). The underlying mechanisms of this organotropism toward specific secondary sites remain poorly understood.

Prognosis remains poor with a median survival ranging from 3 to 27 months after developing brain metastases (3). Treatment options are limited and usually involve multimodality approaches that include radiotherapy, surgery, and sometimes systemic therapy, depending on number of lesions, location, and primary tumor type. Historically, patients with brain metastases were uniformly treated with whole-brain radiotherapy, especially if multiple lesions are present, or surgical resection followed by radiotherapy for a solitary or large symptomatic lesion. More recently, stereotactic radiosurgery has been increasingly used for smaller lesions (<3 cm) in oligometastatic disease, which is commonly defined as up to four brain metastases. CNS-directed systemic treatment options are limited, and patients with brain metastases are commonly excluded from clinical trials, including those investigating novel targeted therapies. Differential responses to systemic treatments between brain metastases and extracranial disease are often observed, particularly with the traditional chemotherapy agents, where systemic disease is adequately controlled while brain disease progresses. This is at least partly explained by inadequate blood–brain barrier (BBB) penetration by systemic therapies (4). Although the BBB is frequently compromised by brain metastases as shown by brain imaging contrast enhancement, the residual BBB permeability limits drug delivery to subtherapeutic concentrations in brain metastases compared with extracranial tumors. With molecularly targeted therapies, there has been improved control of both intracranial and extracranial metastases. In the past decade, significant efforts have been made to characterize the genetic drivers in brain metastases using modern sequencing techniques. Better understanding of the genomic complexity and heterogeneity of brain metastases will lead to improved treatment strategies and research directions. This review focuses on recent advances in genomic profiling of brain metastases and current knowledge of targeted therapies in the management of brain metastases from cancers of the breast, lung, colorectum, kidneys, and ovaries as well as melanoma.

## Genetic Heterogeneity of Cancer

Intratumoral heterogeneity within the primary tumor was shown in a multiregion spatial genetic analysis of four metastatic renal cell carcinoma (5). Genetic heterogeneity between primary and secondary tumors in the same patient was also shown in a study comparing the genetic profiling of 15 primary colorectal cancer and matched liver metastases (6). Similarly, genetic heterogeneity between brain metastases and their corresponding primary tumors was shown recently in a genomic analysis of matched brain metastases, primary tumors, and normal tissue in 86 patients (7). This study demonstrated that brain metastases harbor distinctive potentially actionable mutations not detected in paired primary tumors in 53% of the cases. This finding suggests that this genomic heterogeneity or divergent evolution of brain metastases from primary tumors may also contribute to the disparities in intracranial and extracranial disease response to systemic therapies, which were previously thought to be solely due to the inadequate BBB penetration of systemic drugs. In the same study, it was found that alterations associated with sensitivity to cyclin-dependent kinase (CDK) inhibitors were common in brain metastases (7). These include *CDKN2A* loss and *CDK4/6* amplifications. Alterations in the PI3K/AKT/mTOR pathway were also common as previously reported by others (8–10). Interestingly, brain metastases from different intracranial sites in the same patient shared nearly all of the potentially actionable driver mutations, suggesting that brain metastases are homogeneous within an individual (7). Prospective clinical trials using targeted therapies that cross the BBB are required to demonstrate that these potentially actionable mutations found in brain metastasis samples are indeed targetable.

## Genomic Profiling of Brain Metastases

In the current era of molecularly targeted therapies and precision medicine, choice of cancer treatment has been increasingly tailored according to the molecular or genomic characterization of the cancer the individual has. Identification of genomic alterations specific to brain metastases and targeted therapies against these mutations represent an important research area to improve survival outcomes for patients who develop brain metastases. Given spatial and temporal intratumoral heterogeneity within the same patient, repeated biopsies are often necessary to adequately characterize the somatic genetic alterations in human cancers. Genomic profiling of brain metastases poses a particular challenge as obtaining tissue samples of brain metastases is invasive and often difficult, especially in patients who are poor candidates for brain resections or have tumors in inaccessible sites. In addition, regional lymph node and other distal extracranial metastasis samples are not reliable surrogates for detecting these mutations present in brain metastases (7).

There has been great interest in developing alternative methods of genomic profiling of cancer that are clinically practical and non-invasive. Information collected by these techniques not only help refine systemic treatment decisions but also monitor response to treatment and identify emergent drug-resistant mutations by tracking the evolution of cancer genome to guide further therapy. For example, analysis of circulating tumor DNA (ctDNA) in plasma has been shown to be useful not only in characterizing cancers but also in monitoring disease response to therapy (11–13). However, it was recently shown that tumor DNA was either absent or present in a very little amount in the plasma of patients with primary brain tumors or brain metastases with no or little extracranial disease from solid tumors (12). In the same study, mutations that were present only in the brain metastases and not in the extracranial tumors were more represented in the cell-free ctDNA from the cerebrospinal fluid (CSF) compared to plasma. As seen with plasma ctDNA in previous studies, CSF ctDNA was also observed to change throughout treatments (12). Mutant allelic frequency of CSF ctDNA decreased with tumor response to treatments and increased with progression.

Brain metastases can harbor drug-resistant mutations that result in restoration of signaling downstream of the target kinase, activation of an alternative signaling pathway, or alteration in the drug activating site (14). And these mutations may not be present in the primary tumor or extracranial metastases (7), therefore, genotyping primary tumor or other extracranial disease alone can miss actionable oncogenic driver mutations and targeted therapy opportunities for brain metastases. Routine brain biopsies are invasive with associated risks and may not be feasible in many patients with brain metastases. CSF ctDNA analysis may provide the ability to identify the drug-resistance mechanisms in patients who progressed in the CNS after initial response on targeted therapy without invasive procedures like a brain biopsy. In a recent study, CSF as a source of ctDNA was evaluated by sequencing 341 cancer-associated genes in cell-free DNA isolated from the CSF of 53 patients with brain metastases of solid tumors or primary brain tumors (14). Mutations, which are known to cause drug resistance to oncogenic kinases, were identified in 4 of 12 patients who progressed in the brain whilst on such therapy. Although this needs to be verified in a larger cohort of patients, these findings suggest that CSF ctDNA may be a useful biomarker that facilitates genome-directed treatments to target brain metastases and for monitoring CNS disease on treatment or during surveillance. Moving forward, more studies evaluating ctDNA extracted from CSF should be carried out using next-generation sequencing techniques that are capable of detection all classes of mutations including base substitutions/insertions/deletions, gene fusions, and gene copy number alterations. The fraction of cell-free ctDNA in CSF is higher than in plasma due to the relative absence of background normal DNA in CSF. This allows the detection of somatic mutations even with modest sequence coverage, whereas plasma cell-free ctDNA sequencing requires very deep sequence coverage to achieve sufficient sensitivity for detecting mutations occurring at low allele frequencies. CSF ctDNA analysis appears promising, and there are ongoing studies prospectively evaluating CSF ctDNA as a surrogate for brain metastasis biopsy sample. Furthermore, the use of additional circulating biomarkers, such as exosomes, will need to be prospectively explored.

## Non-Small Cell Lung Cancer (NSCLC)

Non-small cell lung cancer is the most common type of lung cancer, accounting for approximately 85% of all lung cancer cases (15). NSCLC, adenocarcinoma subtype in particular, is the most common cancer to metastasize to the brain (1). The risk of developing brain metastases during the course of disease in patients with treated stage III NSCLC is approximately 30–50% (15). Even in patients with surgically treated early stage (stage I–II) NSCLC, the 5-year actuarial risk of developing brain metastases is reported to be around 10% with the brain being the sole site of failure in 43% of these patients (15, 16). A large retrospective study of 975 patients with stage I–II NSCLC identified younger age, larger tumor size, lymphovascular space invasion, and hilar lymph node involvement to be associated with an increase in the risk of brain metastases in this population (15). Prognosis remains poor even with a multimodality treatment approach of brain metastases. Reported 1-year mortality rate after developing brain metastases ranges from 81 to 90% depending on the initial clinical stage of lung cancer (16). Discovery of NSCLC oncogenic driver mutations and molecularly targeted therapies have significantly improved survivals in the subset of patients with metastatic NSCLC harboring these targetable genomic aberrations.

### Epidermal Growth Factor Receptor (EGFR) Mutations

An activating *EGFR* mutation is present in approximately 10–15% of Caucasians and 40% of East-Asian NSCLC patients (2). When the EGFR status was evaluated in paired brain metastasis and corresponding primary lung tumor samples, discordance rates up to 33% were observed (2). In a recent retrospective, population-based study, there was a higher cumulative incidence of brain metastases in patients with *EGFR*-mutant NSCLC compared to those with *EGFR* wild type (39.2 versus 28.2%) (17). *EGFR* mutations render these tumors sensitive to EGFR tyrosine kinase inhibitors (TKIs) resulting in significantly improved survival outcomes. Several phase III randomized trials compared the efficacy of first-generation EGFR TKIs, gefitinib and erlotinib, and an irreversible ErbB family inhibitor, afatinib, to the platinum-containing combination chemotherapy as first-line treatment in *EGFR*-mutant NSCLC patients. Progression-free survival (PFS) was significantly longer in the EGFR TKI group compared to the chemotherapy group, with hazard ratios for progression or death ranging from 0.38 to 0.58 (18–20).

Prospective data on the efficacy of EGFR TKIs in treating brain metastases from NSCLC are limited. However, currently available data suggest that these agents have CNS activity. *Post hoc* subgroup analyses of combined data from two phase III randomized trials of first-line afatinib in *EGFR*-mutant NSCLC patients, which allowed patients with asymptomatic brain metastases to enroll, showed survival benefit from treatment with afatinib compared to chemotherapy (21). PFS (8.2 versus 5.4 months) and objective response rate (ORR) (70–75 versus 20–28%) were significantly better with afatinib than chemotherapy. Pretreated patients with brain metastases may also benefit from afatinib as suggested by a report of 31 patients, of whom 35% had an objective response with an overall disease control rate of 66% (22). Other retrospective and small phase II studies also showed survival benefit from first-generation TKIs in patients with *EGFR*-mutant NSCLC that had metastasized to the brain (23–26). In these studies, ORR was 58–83%, and median PFS was in the range of 7–15 months.

The majority of patients who had initial response to EGFR TKIs had disease progression due to an acquired resistance within 1–2 years (27). The development of an additional *EGFR* mutation, *EGFR* T790M, is responsible for approximately 60% of this clinically observed acquired resistance (28). A third-generation T790M mutant specific EGFR TKI, osimertinib, has been shown to be highly active in patients who have progressed during prior therapy with EGFR TKIs due to T790M mutation (27). Moreover, a recent preclinical study in animal models demonstrated superior penetration of the BBB with osimertinib than gefitinib or afatinib and sustained tumor regression in an *EGFR*-mutant mouse brain metastasis model (29).

Together, positive CNS activity observed with these EGFR TKIs suggests that incorporation of EGFR TKIs in the treatment of asymptomatic brain metastases from *EGFR*-mutant NSCLC appears reasonable and these agents may be utilized upfront and radiation therapy reserved until progression to avoid radiation related neurotoxicity. Prospective evaluations of EGFR TKIs in active brain metastases and sequential approaches with brain radiotherapy are warranted.

### Anaplastic Lymphoma Kinase (ALK) Rearrangement

A fusion gene comprising portions of echinoderm microtubule like protein 4 (*EML4*) and *ALK* genes with resultant chimeric protein with constitutive kinase activity is found in approximately 2–7% of NSCLC (30, 31). Since its first discovery in 2007, there have been rapid advances in the development of several TKIs targeting this genomic aberration. A first-generation ALK TKI, crizotinib, was shown to significantly improve PFS and ORR in both first- and second-line settings in patients with *ALK*-rearranged NSCLC, compared to chemotherapy (32, 33). For example, in a phase III randomized trial of first-line crizotinib, PFS was 10.9 with crizotinib compared to 7 months with chemotherapy and ORR was 74 and 45%, respectively (33). Despite initial response, drug resistance invariably develops. Next-generation ALK TKIs, including ceritinib and alectinib, have been shown to be effective in patients with crizotinib-resistant disease (34, 35).

Brain metastasis is a relatively common complication of *ALK*-rearranged NSCLC, especially at progression. In a retrospective study, incidence rates of brain metastases were 23.8% at diagnosis, 45.5% at 2 years, and 58.4% at 3 years (36). Crizotinib has been shown to be effective in treating brain metastases, at least initially, as suggested by a large retrospective pooled analysis of patients with asymptomatic brain metastases treated with this agent on the PROFILE 1005 and 1007 trials (37). In this study, an intracranial disease control rate was 56% at 12 weeks in patients with previously untreated brain metastases with a median time to CNS progression of 7 months. In a phase III randomized trial of first-line crizotinib versus platinum containing chemotherapy, patients with stable treated brain metastases were allowed to enroll (38). Intracranial efficacy assessment was part of this study. This study showed that among patients with brain metastases CNS disease control rate was significantly higher with crizotinib at 12 weeks (85 versus 45%), and PFS was also significantly longer with crizotinib (9 versus 4 months). Recently two phase II trials of alectinib have shown remarkable CNS activity against brain metastases with CNS response rates up to 75% and median CNS disease response durations of 10–11 months (39, 40). Interestingly, the cumulative CNS progression rate at 12 months was lower than the cumulative non-CNS progression rate (24.8 versus 33.2%) (40).

### Immunotherapy

Recently, there has been a significant development in immunotherapy to treat various types of cancer including NSCLC. Immune checkpoints, which exist to suppress immune response to protect against detrimental autoimmunity and inflammation, can be co-opted by tumors. Engagement of programmed death 1 (PD-1) receptor on activated T cells by programmed death ligand 1 (PD-L1) on tumor cells leads to T-cell inactivation, which in turn results in immune tolerance and subsequent progression of tumor. PD-1 inhibitors, including nivolumab and pembrolizumab, have shown to significantly improve ORR and survival outcomes in patients with metastatic NSCLC without other targetable mutations such as *EGFR* or *ALK* mutations. Both pembrolizumab and nivolumab were superior to docetaxel in previously treated patients (41, 42). Pembrolizumab was shown to be superior to platinum-containing doublet chemotherapy as first-line therapy in patients with NSCLC with more than 50% of tumor cells staining positive for PD-L1 (43). In another first-line trial, pembrolizumab combined with carboplatin and pemetrexed in PD-L1 unselected NSCLC patients was better than chemotherapy alone (44). These trials, however, excluded patients with active brain metastases. An early analysis of an ongoing trial (NCT02085070) investigating the activity and safety of pembrolizumab in NSCLC or melanoma patients with untreated or progressive brain metastases showed encouraging results (45). In this analysis, 6 (33%) of 18 patients with NSCLC had brain response that was durable. There were no grade 3 or 4 neurological toxic effects.

### Squamous Cell Carcinoma

Squamous cell carcinoma accounts for approximately 20% of all lung cancers. A genomic analysis of primary squamous cell lung cancer in 79 patients showed that *PI3K* aberrant tumors had significantly worse overall survival (OS) (8.6 versus 18.8 months) and a higher incidence of brain metastases (27 versus 0%) compared to *FGFR1* aberrant tumors (9). The authors then analyzed six brain metastasis samples by whole-exome sequencing, four of these patients had matched samples of their primary lung cancer. This analysis showed heterozygous loss of *PTEN* in all of the brain metastasis samples with a pattern of gene expression consistent with *PTEN* loss. A deeper genomic analysis in this study demonstrated clonal heterogeneity with a low degree of shared events (as little as 15%) between the brain metastases and their corresponding primary lung tumor. Further studies examining the exact role of aberrant PI3K pathway in the development of brain metastases and potential targeted therapy are warranted.

## Breast Cancer

Breast cancer is the most common cancer in women and second most common cancer to metastasize to the brain. The exact incidence of brain metastases from breast primary in the current era of modern therapies is unknown; however, a 10-year cumulative incidence of CNS relapse in patients presenting with early stage breast cancer is estimated to be around 4–5% (46, 47). Triple receptor negative disease, basal-like subtype, and human epidermal growth factor receptor 2 (HER2) positive breast cancer have an increased risk of developing brain metastases (46, 48). Early (<2 years after primary diagnosis) occurring brain metastases from breast primary are associated with early onset tumor, negative estrogen receptor (ER) status, HER2 overexpression, and triple receptor negative tumor (49). In comparison, many late occurring brain metastases are from ER positive primary tumors (50). Survival outcomes differ between different subtypes of breast cancer. The median OS after developing brain metastases was longer in patients with HER2-positive breast cancer at 11.5 months compared to luminal HER2 negative breast cancer at 9.3 months and triple receptor negative breast cancer at 4.9 months in a retrospective study of 1,256 patients (51).

Divergence of brain metastases from primary breast tumor has been suggested by several studies, in particular with regards to hormone receptor status. For example, retrospective studies reported a loss of hormone receptor expression in brain metastases compared to their corresponding breast primaries (52, 53), highlighting the importance of multisite tumor characterization, if clinically feasible, in the treatment decision making process. Molecular profiling of paired brain metastases and corresponding primary breast tumors by whole-exome sequencing revealed that brain metastases harbored genomic aberrations in the CDK pathway and PI3K/AKT/mTOR pathways, many alterations of which were not detected in the corresponding primary tumor (7).

A bioinformatics screen of genome-wide breast tumor methylation data available at The Cancer Genome Atlas (TGCA) and analysis of 11 pairs of primary breast tumors and their corresponding brain metastases identified three genes, *GALNT9, CCDC8*, and *BNC1*, that were frequently methylated (55, 73, and 71%, respectively) and silenced in brain metastases (50). Of these, *GALNT9* and *BNC1* were infrequently methylated in primary breast tumors, suggesting that they may be metastasis virulence genes and dysregulation of these occur as late events involved in brain colonization of these cancer cells. GALNT9, which is expressed most abundantly in the brain, plays an important role in *O*-glycosylation and, thereby, in cell adhesion and cell–cell communication. Cancer cells with aberrant *GALNT9* that reach the brain are perhaps favored to proliferate in the new microenvironment through dysregulated cell–cell interaction. *BNC1* encodes a zinc finger transcription factor that is involved in expression of a broad range of genes. Further studies are required to better understand the roles these mutations play in the development of brain metastases and to determine if they represent therapeutic targets.

### HER2 Overexpression

Human epidermal growth factor receptor 2 protein overexpression and/or *HER2* oncogene amplification is found in approximately 20% of all breast cancer patients (54). HER2 overexpression is associated with an increased risk of recurrence and death in the absence of adjuvant HER2-directed therapy, and it predicts response to anti-HER2 therapies. HER2-positive breast cancer carries an increased risk of brain metastases, and approximately 30–50% of patients with HER2-positive breast cancer will develop brain metastases during the course of disease (55), with a cumulative incidence of around 12% at 10 years and 14% at 15 years (46, 56). The propensity of HER2-positive breast cancer to metastasize to the brain may be related to the improved survival of patients with HER2-directed therapy, the limited CNS penetration of HER2-directed agents, and the neuro-tropism of HER2-positive breast cancer (57). HER2-directed agents, including trastuzumab, pertuzumab, lapatinib, neratinib and T-DM1, significantly improve PFS and OS of patients with HER2-positive breast cancer. However, in a retrospective study, 24% of 182 patients with HER2-positive primary breast cancer had HER2 negative metastatic disease, and this discordance was associated with decreased survival (58). If feasible, HER2 status should be repeated in the metastatic disease, including brain metastasis, at relapse.

Trastuzumab, a monoclonal antibody against the HER2 receptor, has limited CNS activity due to its inability to cross the intact BBB. However, there is some evidence of an improved CNS penetration of trastuzumab after disruption of the BBB by radiation therapy. For example, a pharmacokinetic study showed that the ratio of the CSF to plasma levels of trastuzumab in patients with brain metastasis improved significantly from 1:420 before radiotherapy to 1:76 after radiotherapy (59). In patients with concomitant leptomeningeal carcinomatosis, the CSF to plasma ratio after radiotherapy was 1:49. Pertuzumab, another monoclonal antibody against the HER2 receptor, has a significant synergistic antitumor activity in combination with trastuzumab and docetaxel as shown in a randomized phase III placebo-controlled trial of pertuzumab, the CLEOPATRA trial (60). In exploratory analyses of this trial, the median time to development of brain metastases as first site of disease progression was significantly longer in the pertuzumab arm compared to the placebo arm (15.0 versus 11.9 months), and the median OS was 34.4 months in the pertuzumab arm, compared to 26.3 month in the placebo arm (61). Pertuzumab with high-dose trastuzumab in HER2-positive breast cancer patients with brain metastases after radiotherapy is currently under clinical evaluation in a phase II trial (NCT02536339). There is also an ongoing trial of intrathecal pertuzumab and trastuzumab in patients with new untreated asymptomatic or low symptomatic brain metastasis in HER2-positive breast cancer (NCT02598427). Lapatinib is a TKI that acts against both HER2 and EGFR receptors. It has been shown that this agent is able to cross the BBB. In the presence of brain metastases, the brain to plasma concentration of lapatinib was higher at 26% compared to the normal brain parenchyma where the concentration was low at 1.3–2.8% (62). However, the intracranial response to lapatinib alone has been shown to be low at 3–6% (63, 64). In a multicenter phase II study, 45 patients with previously untreated brain metastases from HER2-positive breast cancer were treated with lapatinib and capecitabine combination (55). The addition of capecitabine to lapatinib resulted in an encouraging intracranial response rate of 66% with a median time to intracranial progression of 5.5 months.

### PI3K/AKT/mTOR Pathway

Actionable mutations in PI3K/AKT/mTOR pathway are frequent in breast cancer brain metastases (7). The addition of everolimus, an mTOR inhibitor, to an aromatase inhibitor in patients with hormone receptor positive metastatic breast cancer and to trastuzumab and vinorelbine in patients with HER2-positive breast cancer led to improved survival outcomes in randomized placebo-controlled phase III trials (65, 66). Everolimus crosses the BBB as shown in patients with primary brain tumors. The role of everolimus in the management of patients with breast cancer brain metastases is currently being investigated in clinical trials (NCT01305941, NCT01783756).

### Cyclin D-Cyclin-Dependent Kinase 4/6-INK4-Rb Pathway

Activation of cyclin-dependent kinase 4 and 6 (CDK4 and CDK6) by cyclin D leads to cell proliferation by initiating transition from the G1 phase to the S phase in cell cycle *via* phosphorylation of retinoblastoma protein (Rb). Alterations in this pathway are frequent in various cancer types. CDK inhibitors, such as palbociclib, abemaciclib, and ribociclib, have shown high efficacy in patients with hormone receptor positive breast cancer (67). Good CNS penetration of abemaciclib was recently shown (68). There are ongoing trials investigating these agents in patients with breast cancer brain metastases (NCT02896335, NCT02774681, NCT02308020).

## Melanoma

Melanoma is the third most common malignancy to metastasize to the brain. It is estimated that at least 50% of patients with stage IV melanoma will develop brain metastases during the course of disease (69). With supportive care alone, the median survival from developing brain metastases is only around 2 months (70).

### Mitogen Activated Protein Kinase (MAPK) Pathway

About half of melanoma patients have an activating mutation in *BRAF*, an oncogene involved in the MAPK pathway that is a key regulator of cell growth, division, and differentiation (71). The most common *BRAF* mutation is the substitution of valine for glutamic acid (VAL600Glu or V600E) accounting for approximately 90% of all *BRAF* mutations seen in melanoma. *BRAF* V600E mutant melanoma tends to be more aggressive. BRAF inhibitors, including dabrafenib and vemurafenib, significantly improve survival in patients with metastatic *BRAF* mutant melanoma (72, 73). In a multicenter phase II trial, 172 patients with *BRAF* mutant melanoma with at least one asymptomatic brain metastasis were treated with dabrafenib (74). Patients were divided into two cohorts: those with and without previous local treatments for brain disease. Dabrafenib was active in both cohorts with ORR of 39.2% in patients without previous local therapy and 30.8% in those with previously treated brain metastases and median OS of 33 and 31 weeks, respectively. A retrospective study of 27 patients treated with vemurafenib for their *BRAF* mutant melanoma that had metastasized to the brain reported intracranial ORR of 50% and extracranial ORR of 71% (75). The median intracranial PFS was 4.6 months, and median OS was 7.5 months. Next-generation sequencing analysis of poorly responding brain metastases revealed co-occurring mutations in genes predicted to activate the PI3K/AKT pathway.

MEK kinase, which is downstream of BRAF, is activated by CRAF or members of the PI3K pathway as an escape mechanism from BRAF inhibition. When BRAF inhibitors were combined with MEK inhibitors, the efficacy was further improved in patients with extracranial disease, as evidenced by prolonged PFS and OS shown in phase III trials (76–78). This approach of dual BRAF and MEK inhibition therapy in patients with brain metastases is currently being evaluated in clinical trials (NCT02039947, NCT02537600).

### PI3K/AKT/mTOR Pathway

A molecular analysis of 16 matched pairs of melanoma brain metastases and extracranial metastases showed that activation of the PI3K/AKT/mTOR pathway was enriched in the brain metastases (8). Preclinical cell culture and animal studies of a PI3K inhibitor, BKM120, demonstrated that this agent inhibited the growth of cell lines derived from melanoma brain metastases with inhibition rates of up to 80% and potently induced apoptosis and significantly inhibited the tumor growth of human brain metastatic melanoma cells in the brain of nude mice (79). These findings suggest the potential of PI3K inhibitors as adjunct targeted therapy in the treatment of advanced melanoma that has metastasized to the brain.

### Immunotherapy

There has been dramatic improvement in survival of patients with advanced melanoma in recent years with the development of modern immunotherapy including cytotoxic T lymphocyte antigen-4 (CTLA-4) inhibitors and programmed death-1 (PD-1) checkpoint inhibitors. CTLA-4 plays an important role in the regulation of immune activation and tolerance (80). Its signaling inhibits T-cell activation. Ipilimumab is an inhibitor of CTLA-4 that was shown to improve OS in patients with metastatic melanoma compared to a peptide vaccine in a phase III trial (81). A phase II study of ipilimumab was conducted in 72 melanoma patients with brain metastases (82). The disease control rate was 24% in patients who were asymptomatic from their brain disease and were not on corticosteroid treatment. One- and two-year survival rates were 31 and 26% in this steroid-independent cohort. Interaction between PD-1 receptor and PD-ligand 1 (PD-L1) inhibits cytotoxic T-cell activity. PD-1 inhibitors, including nivolumab and pembrolizumab, have resulted in significantly improved survival outcomes in patients with advanced melanoma (83, 84). Pembrolizumab is currently under clinical evaluation in patients with brain metastases from melanoma (NCT02085070). Interim analysis from a phase II trial reported an intracranial ORR of 22% (45). In a randomized phase III trial, nivolumab and ipilimumab combination was shown to be superior to either drug alone in patients with advanced melanoma without brain metastases (85). Median PFS was 11.5 months in the combination arm compared to 6.9 months in the nivolumab alone arm and 2.9 months in the ipilimumab alone arm. There are ongoing trials evaluating the dual CTLA-4 and PD-1 inhibition therapy in melanoma patients with brain metastases (NCT02320058, NCT02621515).

Table 1 summarizes actionable mutations in brain metastases of NSCLC, breast cancer and melanoma, and their targeted therapies discussed earlier.

**Table 1:** Overview of actionable mutations in brain metastases of non-small cell lung cancer, breast cancer and melanoma, and potential targeted therapies.

Cancer type	Mutation	Targeted therapy	Objective response rate (%)	Progression-free survival (months)	Reference or clinicaltrials.gov number
Non-small cell lung cancer	Activating *EGFR* mutation	First-generation EGFR TKIs: gefitinib, erlotinib	58–83	7–15	(23–26)
		ErbB family inhibitor: afatinib	70–75	8	(21)
	*EGFR* T790M	T790M specific EGFR TKI: osimertinib	NA	NA	(29)
				Superior blood–brain barrier penetration than gefitinib or afatinib demonstrated in preclinical study	
	*ALK* rearrangement	First-generation ALK TKI: crizotinib	56–85	7–9	(37, 38)
		Second-generation ALK TKI: alectinib	75	10–11	(39, 40)
	(No specific mutation)	PD-1 inhibitor: pembrolizumab	NA	NA	NCT02085070 (45)
			Early analysis showed 33% intracranial response rate		

Breast cancer	HER2 overexpression/*HER2* oncogene amplification	Dual anti-HER2 inhibition: pertuzumab plus trastuzumab	NA	NA	Intravenous: NCT02536339
					Intrathecal: NCT02598427
		HER2/EGFR TKI: lapatinib (in combination with capecitabine)	66	5.5	(55)
	Mutation in PI3K/AKT/mTOR pathway	mTOR inhibitor: everolimus	NA	NA	NCT01305941
					NCT01783756
	Mutation in CDK4/6 pathway	CDK inhibitors	NA	NA	NCT02896335
					NCT02774681
					NCT02308020

Melanoma	Activating *BRAF* mutation	BRAF inhibitors: dabrafenib	39	16	(74)
		Vemurafenib	50	4.6	(75)
		Dual BRAF/MEK inhibition: dabrafenib + trametinib	NA	NA	NCT02039947
		Vemurafenib + cobimetinib	NA	NA	NCT02537600
	Mutation in PI3K/AKT/mTOR pathway	PI3K inhibitor: BKM120	NA	NA	(79)
				Efficacy demonstrated in preclinical study	
	(No specific mutation)	CTLA-4 inhibitor: ipilimumab	24	2.7	(82)
		PD-1 inhibitor: pembrolizumab	NA	NA	NCT02085070
		Dual CTLA-4/PD-1 inhibition: ipilimumab + nivolumab	NA	NA	NCT02320058
					NCT02621515

*EGFR, epidermal growth factor receptor; TKI, tyrosine kinase inhibitor; ALK, anaplastic lymphoma kinase; PD-1, programmed death 1; HER2, human epidermal growth factor receptor 2; PI3K, phosphoinositide 3-kinase; mTOR, mammalian target of rapamycin; CDK4/6, cyclin-dependent kinase 4 and 6; CTLA-4, cytotoxic T lymphocyte antigen-4; NA, not available*.

## Colorectal Cancer

While colorectal cancer is the third most common cancer in men and second in women, brain metastases are relatively rare with an incidence rate of 1.55% (86). A systematic review and pooled data analysis from 16 relevant studies showed that rectal location was associated with an increased risk of brain metastases compared to other colon locations. The presence of pulmonary metastases was also associated with an increased risk. The incidence of brain metastases from colorectal cancer may be increasing as a result of improved systemic therapy and survival of patients. The prognosis is poor with a reported median OS of 2.6–7.4 months after developing brain metastases (86).

### *RAS* Mutation

*KRAS* and *NRAS* mutations are the most extensively investigated somatic mutations in metastatic colorectal cancers, and they predict tumor resistance to anti-EGFR therapy, such as cetuximab and panitumumab. There have been several studies examining these mutations in patients with brain metastases from colorectal cancer. A retrospective analysis of 918 patients with metastatic colorectal cancer who were genotyped for *RAS* mutations showed a significantly higher cumulative incidence of brain metastases at 2 years in patients with *RAS* mutation compared to those without (1.4 versus 0.2%) (87). Nearly two-thirds of the brain metastasis cases occurred in patients with *RAS* mutant colorectal cancer. Currently, there are no RAS inhibitors available for clinical use.

### *BRAF* Mutation

*BRAF* activation mutations (most commonly V600E) occur in less than 10% of metastatic colorectal cancer and are associated with poorer survival. *BRAF* V600E mutations confer resistance to anti-EGFR therapy. To date, no clear association between *BRAF* mutation and development of brain metastases has been observed (88, 89). A clinical characterization of 524 metastatic colorectal cancer patients with known *BRAF* mutation status, where 57 patients had *BRAF* mutation, found no difference in the incidence of brain metastases between patients with *BRAF* mutant tumors and those with wild-type tumors (88).

### *PIK3CA* Mutation

*PIK3CA* mutations are found in approximately 10–15% of primary colorectal cancers. In a genomic analysis of colorectal metastases from different sites, *PIK3CA* mutation frequency was higher in brain and lung metastases compared with liver metastases (23.9% in brain, 20% in lung, 7.7% in liver) (89) Another study also found an association between *PIK3CA* mutations and brain metastases with a 2-year cumulative incidence of 1.4%, compared to 0.8% in patients with no *PIK3CA* mutation (87). However, this observation is difficult to interpret as many of those patients with *PIK3CA* mutation and brain metastases also had *RAS* mutations.

## Renal Cell Carcinoma

Approximately 3.5–17% of patients with renal cell carcinoma develop brain metastases (69). Reported median OS after developing brain metastases ranges from 4.1 to 15 months (69). Patients with bone and thoracic metastases were found to have a higher rate of brain metastases compared to those with abdominal metastases (16 versus 2%) (90). An association between the loss of chromosome 9 and brain metastases was also observed (91). Registration-directed clinical trials investigating therapies for angiogenesis, mTOR signaling, and immunotherapy have excluded patients with brain metastases. Patients with brain metastases were included in the European Advanced RCC Sorafenib expanded-access study in which sorafenib, a VEGF TKI, was given to patients with previously treated advanced renal cell carcinoma. It was shown that the sorafenib safety profile in patients with brain metastases was similar to the overall study population (92). In a prospective open-label non-interventional study in a broad population of patients with advanced renal cell carcinoma treated with sorafenib in routine clinical practice, 115 patients (5% of total study population) had brain metastases (93). The median duration of therapy for this subgroup was similar to that of the total study population (7.0 versus 7.3 months), suggesting that sorafenib has activity in the CNS. In a retrospective study, treatment with TKI was associated with an improved median OS from developing brain metastases (94). However, in prospective studies, the response to these drugs has been more modest. A reported median PFS and OS were only 5.3 and 8.2 months, respectively, in patients with brain metastases treated with sunitinib as part of the global expanded-access protocol (95).

Genetic divergence of brain metastases from primary renal cell carcinoma has been shown recently in a genomic study of paired brain and primary tumors (7). In this study, some brain metastases from renal cell carcinoma had *PTEN, PIK3CA*, and *CDKN2A* mutations that were not detected in the corresponding primary tumors. Mutations in PI3K/ATK/mTOR pathway may be potential drivers in brain metastases development and therefore warrant further investigations.

In a randomized phase III trial, the treatment with nivolumab improved survival outcomes compared with everolimus in patients with previously treated advanced renal cell carcinoma (96). Unfortunately, patients with brain metastases were excluded. Clinical trials investigating the role of nivolumab or pembrolizumab in patients with brain metastases is currently underway (NCT02978404, NCT02596035, NCT02886585).

## Ovarian Cancer

Brain metastases from ovarian cancer are very rare with reported incidence rates ranging from 0.29 to 5%; however, the incidence has been rising since the introduction of platinum-based chemotherapy (97). In a recent next-generation sequencing-based genomic analysis of eight brain metastases of primary ovarian cancer, all eight brain metastasis samples harbored mutations in at least one DNA repair gene with seven of eight samples revealing either a *BRCA1* or *BRCA2* mutation (98). Other commonly observed mutations include *TP53, ATM*, and *CHEK2* mutations. These findings suggest that BRCA and DNA repair malfunction may possibly play a role in ovarian cancer metastasizing to the brain. A mutation in *ATM*, a regulator of DNA damage detection and repair *via* phosphorylation of a wide variety of downstream proteins including TP53 and BRCA1, may also play a role in the development of brain metastases (98). In cancers that lack homologous repair capacity due to BRCA1/2 or ATM dysfunction or loss, PARP inhibitors can lead to cancer cell death *via* mechanisms of synthetic lethality (99). PARP inhibitors, olaparib and veliparib, have been shown to have activity in the CNS (100, 101), therefore they represent a potential targeted therapy in these cancers with genetic alterations in genes involved in DNA damage response and repair.

## Conclusion

The incidence of brain metastases is rising as modern cancer therapy is improving the survival of patients with advanced cancer. There is mounting evidence of genomic heterogeneity intratumorally as well as across different metastatic sites. Recently, genomic analyses of brain metastases and matching primary tumors have revealed that brain metastases can harbor actionable driver mutations that are not present in the primary tumors or other extracranial metastases. This genomic divergence of brain metastases from their primary tumors may contribute to the clinically observed treatment response disparities. Cancer genomic analysis using novel and far less invasive approaches, such as cell-free ctDNA in the CSF obtained *via* a lumbar puncture in the outpatient clinics, is very promising in providing critical information required for personalized genomic-directed therapy in patients with brain metastases. Moreover, these new less invasive genomic analysis techniques are more feasible even in patients who are not surgical candidates and can be repeated to determine tumor response to treatment or detect early progression during surveillance. Compared to the traditional chemotherapeutics, next-generation targeted agents appear to be more effective in controlling the CNS disease with better safety profiles. Several brain metastases-dedicated trials of various targeted therapies are currently underway to address the role of these agents in the treatment of CNS disease.

## Author Contributions

All the authors contributed to the manuscript.

## Conflict of Interest Statement

PB has consulted for Merck, Lilly, Genentech, and Angiochem. CH declares no conflict of interest.
